# In Vitro Culture of Avian Primordial Germ Cells: Established Methods and Future Directions

**DOI:** 10.3390/biology14111597

**Published:** 2025-11-14

**Authors:** Jehan Nayga, Elen Gócza, Eszter Várkonyi, Bence Lázár

**Affiliations:** 1 Institute of Genetics and Biotechnology, Animal Biotechnology Department, Hungarian University of Agriculture and Life Sciences, 2100 Gödöllő, Hungary; 2 Agribiotechnology and Precision Breeding for Food Security National Laboratory, 2100 Gödöllő, Hungary; 3 National Centre for Biodiversity and Gene Conservation, Institute for Farm Animal Gene Conservation, 2100 Gödöllő, Hungary

**Keywords:** primordial germ cell, PGC, in vitro culture, bird species, germline chimera

## Abstract

Primordial germ cells (PGCs) are the unipotent precursors of sperm and ova, responsible for transmitting hereditary information across generations. Their ability to be isolated and cultured in vitro has opened new horizons for avian biotechnology, species conservation, and fundamental developmental research. In birds, the unique migratory pattern of PGCs—originating in the epiblast and traveling via the bloodstream to the gonads—enables their collection and manipulation during embryogenesis. Long-term in vitro culture systems have been successfully established in chickens, where defined media allow for stable proliferation and genetic modification. Applications include germline chimeras, generation of transgenic lines, recombinant protein production, and cryobanking of genetic resources. However, translating these advances to other species remains challenging due to interspecies variability in signaling requirements. Recent work in geese, ducks, quails, and zebra finches underscores the need for tailored media formulations and a better understanding of molecular regulation. This review summarizes established techniques, highlights key interspecies differences, and outlines future directions for the standardization and expansion of avian PGC culture systems to support conservation and biotechnology.

## Simple Summary

Primordial germ cells (PGCs) are the reproductive precursor cells that give rise to sperm and eggs. Studying and cultivating these cells in the laboratory offers new opportunities for biotechnology and gene conservation. Bird embryos cannot be easily cloned or cryopreserved; however, avian PGCs can be used as an alternative tool. In chickens, researchers have already developed efficient methods to isolate, grow, and genetically modify PGCs, which can then be used to produce offspring carrying desired traits. This technology is now being adapted to other bird species, including geese, ducks, quails, and zebra finches. Further research into species-specific culture conditions will be essential to apply PGC-based methods for biodiversity conservation and sustainable poultry production.

## 1. Introduction

Primordial germ cells (PGCs) represent the foundational cell lineage responsible for transmitting genetic and epigenetic information to subsequent generations. As such, understanding their fundamental biology and learning their manipulation in vitro are valuable in advancing avian biotechnology, genetic resource conservation, and developmental biology. A characteristic feature in vertebrates is the extragonadal origin of PGCs, meaning they appear in a region of the early embryo remote from the site of the future gonads [1]. Therefore, there is a complex and precisely regulated migration, and the method of migration of avian PGCs is distinct from that of many other vertebrates. Originating from the central region of the epiblast, PGCs translocate to an extraembryonic region known as the germinal crescent [2,3]. From there, in a process unique to avians, they passively enter newly forming blood vessels, initiating a circulatory phase during specific Hamburger and Hamilton (HH) stages (e.g., HH13–HH17) [4]. After traveling throughout the embryonic bloodstream, they eventually exit the vasculature and actively migrate to colonize the developing gonads following chemotactic signals by HH28–HH30 [5]. Generally, the chemotactic interaction between stromal cell-derived factor 1 (SDF1) and its receptor CXCR4 directs PGC migration in vertebrates [6,7,8,9,10]. In chickens, CXCR4 expression in PGCs and attraction to ectopic SDF1 confirm this pathway’s importance [11,12], while the knockout of *CXCR4* reduces migration efficiency [13]. Additional mechanisms have been proposed as well. One study proved PGC stiffness aids the occlusion near the gonads [14] and another showed platelet-derived growth factor signaling involvement [15]. The vascular migration in birds stands in contrast to the amoeboid, tissue-based migration of PGCs observed in mammals [16].

The ability to intercept PGCs during their migration and establish stable in vitro cultures has unlocked a range of powerful biotechnological applications, as these cells serve as a direct and efficient conduit to the avian germline [17,18,19].

This is particularly valuable in light of the several challenges associated with avian sperm and egg cryopreservation, cloning and the characteristics of their reproductive biology. For sperm cryopreservation, a major issue is the reduced viability and fertility caused by ice crystal formation, osmotic stress, and oxidative damage during freezing and thawing, which can impair sperm function [20]. Additionally, optimal cryopreservation protocols vary between species, requiring species-specific approaches [21,22]. An additional limiting factor in birds is that the female is the heterogametic sex (with ZW sex chromosomes), so methods based on freezing of sperm only are not able to preserve the information stored in the W sex chromosome and mitochondrial DNA. Furthermore, early-stage avian embryos are not easily accessible in the female’s reproductive tract due to protein, shell membranes and the hard egg shell being synthesized and covering the forming embryo. Therefore, traditional techniques from mammalian biotechnology using early embryos, such as embryo cryopreservation and cloning (somatic cell nuclear transfer), are not possible, and it is not likely it would be feasible in the future.

**Figure 1:**
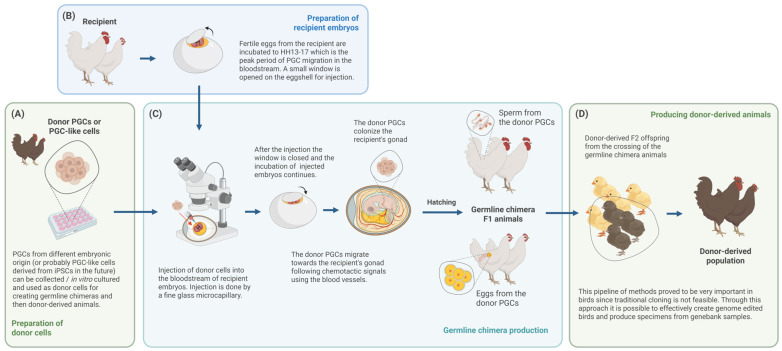
Production of donor-derived birds through germline chimeras. (**A**) Donor PGCs can originate from various sources and they can be in vitro cultured, genome-edited and/or cryopreserved. (**B**) Before injection, recipient embryos are incubated to HH13–17, which is the peak of PGC migration; then, a small hole is opened in the eggshell for manipulation. (**C**) Donor PGCs are injected into the bloodstream of recipient embryos, where they migrate towards the gonads and eventually colonize them, creating germline chimera animals. (**D**) With the crossing of the F1 generation, donor-derived offspring can be produced. (Created in https://BioRender.com).

To fight these challenges, a set of methods has been developed around primordial germ cells. In chicken, once isolated from the donor embryo, PGCs can be propagated for extended periods in vitro, enabling the generation of large cell populations for cryopreservation or precise genetic manipulation [23,24,25]. These PGCs can then be reintroduced into surrogate host embryos to efficiently produce donor-derived offspring [26,27]. The outline of this process is shown in Figure 1.

Despite the increasing number of studies, the development of efficient and reliable methods for culturing PGCs in species beyond chickens remains an ongoing challenge. The main limiting factor in the application of PGCs in gene conservation and fundamental research is that they need different formulations of culture media for proliferating, sometimes even within species [28].

This review aims to synthesize current knowledge on applications and sources of avian primordial germ cells, compare various in vitro culture techniques, and identify future research directions.

## 2. Applications of PGCs

**Figure 2:**
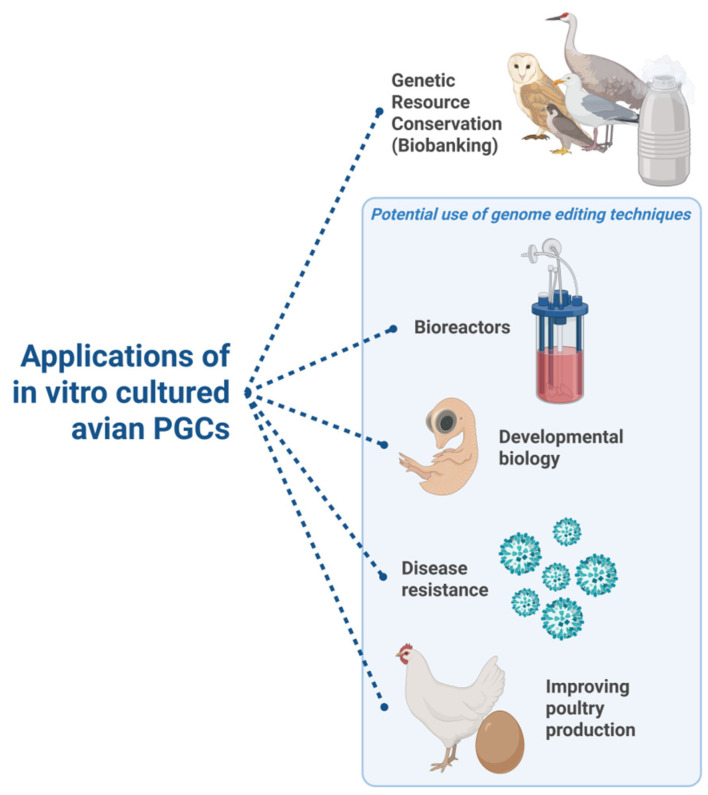
Applications of in vitro cultured PGCs. The possibilities are diverse, ranging from conservation of genetic material through fundamental research of embryo development to aiding animal husbandry. The combination of in vitro culture and genome editing is particularly important and powerful for most applications. (Created in https://BioRender.com).

In this section, representative examples are presented that illustrate the broad and multidisciplinary applications of avian primordial germ cells (PGCs) (Figure 2).

PGCs have a great value in the conservation of genetic resources. Culturing and cryobanking of these cells provides an innovative method to preserve the complete genomes of rare, endangered, or specialized poultry breeds, mitigating the risk of extinction and irreversible genetic loss [29,30]. This approach has been proven viable for preserving indigenous breeds (Partridge color Hungarian chicken [24]); experimental flocks [26] or blue layer hybrids (Araucana crossbreeds) [31]. Efforts toward conserving wild species have also been presented (red junglefowl and several indigenous pheasant species, including the bar-tailed, kalij, Siamese fireback, and silver pheasants [32]).

Cryobanking of avian PGCs is the most effective method for preserving both male and female genetic material. Researchers in several countries have initiated gene-banking programs using PGCs to conserve the genetic resources of local chicken breeds. Such efforts have been implemented in Thailand [33], China [34], and Hungary [24].

The improvement of the 3D, scalable production of avian cell lines is not just promising for therapeutic applications, such as producing recombinant proteins, but also has the potential to generate large-scale cultures of PGCs for the in vitro meat or ‘clean meat’ industry [35]. In addition to PGCs, chicken embryonic stem cells (cESCs) are also among the primary cell types explored for cultivated meat production. Derived from the blastoderm of fertilized eggs at embryonic stage X, cESCs exhibit pluripotency, allowing indefinite self-renewal and differentiation into cell types from all three germ layers [36,37]. Their ability to generate muscle, adipose, and connective tissues makes them promising for cellular agriculture applications [38,39].

Avian PGCs serve as the principal cellular vector for generating transgenic and genome-edited birds, offering a highly efficient alternative to direct manipulation of the avian zygote. This strategy circumvents the significant biological barrier posed by the avian reproductive system, where the zygote is inaccessible within the hen’s reproductive tract and is already a multicellular blastoderm surrounded by a large yolk upon being laid. The capacity to isolate, culture, and genetically manipulate PGCs in vitro before introducing them into a host embryo is the foundation of modern avian transgenesis [25,40]. The applications and possibilities of these tools for altering the avian genome are diverse.

For instance, transgenic chickens can be engineered to function as bioreactors, producing high-value recombinant proteins in the whites of their eggs. Using PGCs as the vector for gene transfer, researchers have successfully created chickens that secrete therapeutic proteins, such as human interferon-α 2a (hIFNα 2a) and hepatitis B virus surface antigen (HBsAg), under the control of an oviduct-specific promoter [41]. Gene-edited chickens that synthesize human 1gG1 Fc in the liver and secrete α-2,6 sialylated and low-fucosylated human IgG1 Fc (rhIgG1 Fc) into serum and egg yolk were also created. The egg yolk as a bioreactor expresses recombinant human IgG1 Fc [42].

Other existing genome-edited chickens produce recombinant human adiponectin [43,44], human interferon beta [44], and functional human erythropoietin (hEPO) [45], and recombinant monoclonal antibodies (mAb) in egg whites [46].

The capacity to culture, genetically modify, and reintroduce avian PGCs has transformed the chicken into a premier model organism for functional genomics and developmental biology. These techniques permit the precise investigation of gene function in vivo, overcoming technical barriers in avian transgenesis and enabling researchers to answer fundamental biological questions and create new research tools. To resolve how male (ZZ) and female (ZW) birds compensate for differences in Z-linked gene dosage, researchers used CRISPR/Cas9 in cultured PGCs to knock out miR-2954 on the Z chromosome. The experiment revealed that miR-2954 is essential for male embryonic survival by preventing the lethal upregulation of dosage-sensitive Z-linked target genes, thereby uncovering a unique dosage compensation system in which a miRNA is critical for male viability [47]. Additionally, CRISPR/Cas9 editing in cultured PGCs successfully generated Z chromosome knock-in models that enable sex identification of chickens using fluorescence detection during embryogenesis [48].

Generating transgenic avian species using cultured PGCs and genome-editing tools for developmental biology and neuroethological studies has become an emerging approach to understanding the complexity of biological development and behavioral processes. Such studies are conducted in songbirds [49] and chickens [50,51].

Transgenic chickens have also been produced through CRISPR/Cas9-mediated in vivo transfection of PGCs within embryos. This approach provides a more direct alternative to conventional methods that require extended in vitro culture of PGCs prior to genome modification. Specifically, the Interferon- α/β Receptor 1 (IFNAR1) and Interleukin 1 receptor, type I (IL1R1) genes were targeted [52].

Additionally, a transgenic chicken line was successfully generated through Tol2-mediated genomic integration via in vivo electroporation, yielding a double fluorescent reporter line that distinctly labels limb mesenchymal cells and apical ectoderm ridge (AER) cells [53].

The combination of PGC culture with genome editing tools like CRISPR/Cas9 and TALENs allows for precise gene knockouts, insertions, and modifications. For instance, disease resistance can be engineered against major avian pathogens, offering a definitive and heritable solution to pressing veterinary challenges. An elegant example of this approach is the successful creation of chickens resistant to Avian Influenza Virus [54], and avian leukosis targeting the avian leukosis virus subgroup J (ALV-J) [55].

Lastly, PGC technology can help the poultry industry by boosting productivity and feed efficiency, and even by producing heat-stress-resilient chickens [56,57].

## 3. Sources of Avian PGCs

**Figure 3:**
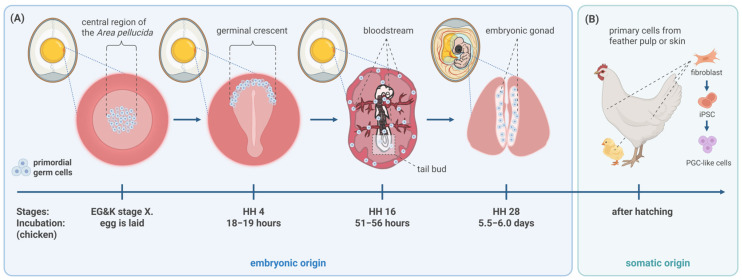
Origin and migration of PGCs and their potential sources for in vitro culture. (**A**) The developmental pathway of avian PGCs is distinct among vertebrates. Avian PGCs originate in the central area of the blastoderm at EG&K stage X. They subsequently migrate to an extra-embryonic region known as the germinal crescent by HH stage 4. As the embryonic vascular system forms, PGCs enter the bloodstream around HH stage 11−12 and circulate throughout the embryo, with their concentration peaking at HH stage 13−17. Finally, they exit the vasculature and actively colonize the developing gonads by HH stage 28−30. These are also the periods when it is possible to isolate them from the embryo by dissection (EG&K X, HH 4 and HH 28) or blood collection (HH 16). (**B**) Recently, there has been a new approach to generating PGC-like cells via somatic cell sources. Primary cell cultures from skin or feather pulp (FFC) can be established, and then reprogramming them to iPSCs opens the way for creating PGC-like cells, which can be used as donor cells. Although offspring have not been generated from such cells with further improvement, it can be a valuable alternative. (Created in https://BioRender.com).

Avian PGCs can be isolated from several locations in the developing embryo at different timepoints, with the choice of source often depending on the developmental stage and desired cell yield (Figure 3).

PGCs can be collected with microsurgery from early stages. The central zone of the Area pellucida from the blastoderm (EG&K stage X) can be collected, which contains PGCs (approx. 30 PGCs) and blastodermal cells too [3]. This mixed cell population is capable of making germline and somatic chimeras, but typically, the cell number and hence the efficiency are low [58,59,60,61]. Chimera production was also reported after short-term culture [62,63,64] and cryopreservation [65] of PGCs isolated from the stage X blastoderms. The germinal crescent region of stage HH 5–11 embryos can also be dissected, and with the isolated cells (approx. 100–150 PGCs), germline chimeras were produced [66,67]; however, in vitro culture of these cells has not been reported [64]. In comparison, these isolation methods require more skill or result in fewer PGCs than the techniques below and, therefore, are nowadays rarely applied.

On the other hand, circulating PGCs (cPGCs) are a more widely used option, and several studies demonstrated successful culture establishment and germline chimera production from them [23,24,26,50,68]. These cells are harvested directly from embryonic blood (approx. 200 PGCs) during their transient circulatory phase (e.g., HH13–17 in chicken). This method provides early-stage access, collection is rather quick, and the procedure can be performed without sacrificing the donor embryo. The downside is the lower PGC concentration.

Gonadal PGCs (gPGCs) are isolated from dissociated embryonic gonads after PGCs have completed their migration (e.g., HH28–30 in chicken) [29,69]. The concentration of PGCs is substantially higher in gonadal tissue, which often results in a greater initial cell yield [31], which translates to faster proliferation in vitro. Some studies report favorable results with cultures started from gPGCs [31,70]. Although embryo sacrifice is required using this method.

Novel methods have been developed to isolate PGCs from embryonic tissues post-exsanguination, termed “tissue PGCs” (tPGCs) [32]. A successful application was demonstrated in Japanese quail, where PGCs were efficiently isolated from embryonic tail buds (“tbPGCs”), cultured and PGC migration into the recipient’s gonad was shown [71].

Finally, there is an entirely different approach to acquiring cells with PGC characteristics, even without using embryonic tissues or cells. Thanks to the rapid improvement of methods and protocols on induced pluripotent cell generation, iPSCs of different avian species were generated as well [72]. iPSCs reprogrammed from somatic tissues were reported in the case of wild birds, which opens up huge possibilities for gene conservation [72]. Nowadays, a promising somatic cell source is the feather pulp from which feather follicle cells (FFCs) can be collected and primary fibroblast cultures can be established [73,74]. These fibroblast cells serve as a good starting point for reprogramming. From iPSCs, PGC-like cells can be differentiated [75], which are then suitable for the creation of germline chimeras and, ultimately, in theory, donor-derived animals. In 2021, a group reported that chicken embryonic fibroblast cells could be transdifferentiated to primordial germ cells and then used to generate viable offspring through germline chimeras [76]. In 2023, this article was retracted due to concerns about the analyses of the origin of the produced offspring (no concerns were raised regarding data integrity) [76]. Therefore, as of now, offspring have not been generated from reprogrammed and differentiated cells.

iPSC technology also offers a powerful model to study in vitro gametogenesis by enabling the reprogramming of somatic cells into pluripotent states capable of differentiating into PGCs. This system allows detailed analysis of germline specification and development mechanisms, bridging knowledge gaps between mammalian and avian reproduction. Avian iPSCs, particularly in chickens, have been used to explore cell differentiation pathways and germline transmission, highlighting their potential in reproductive biotechnology and species conservation. However, major limitations remain. These include incomplete understanding of avian-specific reprogramming environments, inefficient induction of functional PGCs, instability of pluripotency, and technical constraints in gene delivery and epigenetic remodeling. Overcoming these challenges is essential to achieving stable, functional iPSC-derived PGCs for avian reproductive applications [77,78].

In conclusion, the iPSC field is challenging, and previous expectations have not yet been met. However, in case such a system based on transdifferentiation will be developed, it will be a very useful addition to the avian biotechnology toolbox.

## 4. Species Where In Vitro PGC Culture Was Established or Researched

The limited number of PGCs available from a single embryo made it necessary to develop methods to expand the cell number in vitro for practical applications. Research in this area was focused on chicken for the first 20 years or so, then made its way towards other species. Adapting PGC culture protocols beyond the chicken serves two purposes: it extends the technology for practical applications and simultaneously explores the evolutionary conservation of the signaling pathways governing germline self-renewal across the avian class.

Furthermore, chickens are *Galloanserae,* which taxon represents only 4% of all bird species, while *Neoaves* holds 95%. Therefore, we need to investigate the PGCs of *Neoaves* species too, since their characteristics might be much more relevant for the majority of birds than what we know from the chicken [79].

An additional consideration for developing in vitro culture for non-chicken species is the recent findings on germline-restricted chromosome (GRC) in songbirds (*Passeri*), which taxon holds half of all birds (~5100 species), including the zebra finch. GRC can be found only in germ cells after it is eliminated from somatic cells during embryonic development, which highlights the importance of PGCs versus the other embryonic or somatic cell types for songbirds [80,81].

Based on the current knowledge, although there are conserved characteristics, each species presents unique biological challenges, and thorough analysis of signaling pathways and gene expression is needed to precisely compose culturing media that support long-term proliferation and germline competency.

This section reviews the methods and results achieved in avian species where in vitro culture has been explored. The collected information is also shown in Table 1.

### 4.1. Chicken

The first demonstration of in vitro culture was made possible by a feeder layer from stroma cells derived from genital ridges of 5-day-old embryos, and it supported PGCs isolated from blood or embryonic gonads for 4–5 days. Cultured PGCs were still able to migrate, colonize and proliferate in recipient chicken embryonic gonads [89].

The first successful long-term cultures were established by van de Lavoir et al. demonstrated that chicken PGCs could be propagated for extended periods, though these initial systems relied heavily on feeder layers of somatic cells, such as STO or BRL cells, and the use of conditioned medium to provide essential, undefined growth factors [82]. Additionally, it was better suited for male PGCs and less optimal for female cells. It was further optimized by showing that the membrane-bound form of chicken SCF (SCF2) aids PGC proliferation [83].

Later on, a transition occurred with the move towards defined, feeder-free culture systems. This shift was enabled by the systematic search for the minimal signaling pathways required for PGC self-renewal, which was the approach needed to replace the undefined factors previously supplied by conditioned medium and feeder cells. Important research by Whyte et al. identified that a combination of FGF, Insulin, and SMAD signaling (activated by either Activin A or Bone Morphogenetic Protein 4, BMP4) was sufficient to maintain chicken PGC proliferation and identity in vitro [23]. In the case of this medium, it was also proven that culturing the PGCs in it does not affect most of the gene expression compared to in ovo PGCs (except for a minor difference in proliferation-related genes) [90].

Next, further optimization of culture media was carried out, including the development of serum-free formulations. For instance, long-term in vitro culture of cPGCs is feasible using a defined three-dimensional culture system (‘3D-FAot medium’). In this serum-free system, researchers used FP003 polymer, FGF2, Activin A, and ovotransferrin to increase cPGC number 17-fold in one week. After one month, cPGCs in 3D-FAot retained their characteristics and functions, stably expressing recombinant fluorescent proteins (EGFP and tdTomato) [35]. Additionally, the use of enhanced medium with Knock-Out Serum Replacement (KSR), which contains a lipid-rich albumin called AlbuMAX, resulted in improved cultivation, derivation, and expansion of cPGCs, making it suitable for long-term culture [84]. Additionally, studies have shown that small molecules like blebbistatin and CHIR99021 could enhance PGC self-renewal by reducing apoptosis, which is another option for media optimization [31,91,92,93].

There are developments in culture equipment as well, for instance, a recent study successfully isolated primordial germ cells from the blood of chicken embryos using a 3 μm cell culture insert in conjunction with chicken embryonic fibroblasts. The process achieved a separation rate of 87.5% with a purity of 95%. The isolated PGCs began proliferating by day 2 in feeder-free cell culture media and exhibited a 37-fold increase in proliferation within two weeks, and kept proliferating after 30 days as well [94].

Although nowadays feeder-free culturing is considered the more advanced technique, there are still improvements on the feeder cell-based approach. In a study, different chicken-derived feeder layers were compared, specifically chicken mesenchymal stem cells (MSCs) from bone marrow, against chicken embryo fibroblasts (CEFs). Notably, the chicken MSC feeder layer outperformed the CEF feeder layer, potentially providing a more conducive microenvironment for cPGCs [95].

While these collective advancements established a well-characterized set of signaling requirements in the chicken model, evidence suggests that even between chicken breeds, there can be differences in nutritional preference for PGCs, resulting in cultures with varied quality [27]. Therefore, in addition to developing a medium for new avian species, it is also important to test and evaluate already established recipes for new chicken breeds.

### 4.2. Goose

Apart from the chicken, the only other avian species with a proven long-term in vitro culture established is the goose. Earlier this year, a study described that, unlike chicken primordial germ cells, the self-renewal of goose PGCs is dependent on the TGF-β family member BMP4 and, conversely, is inhibited by Activin A. Therefore, the culture medium for goose PGCs contains the following components: 4 ng/mL FGF1, 4 ng/mL FGF2, 25 ng/mL BMP4, and 50 μg/mL ovotransferrin (collectively referred to as FBot), along with B27 supplement; 1× vitamin mix, vitamin B12, cholesterol, retinol, and 50 μg/mL IGF-1, all in DMEM supplemented with 0.075 mM calcium chloride. While FGF, BMP and Insulin signaling are shared characteristics with chicken PGCs, the addition of cholesterol and lower calcium levels were also required for goose long-term in vitro growth. Here, the medium is replaced every 3 days while the cells are maintained under proliferative conditions in a 48-well plate. Goose PGCs were kept in culture for 3–4 months to prove long-term propagation while keeping their self-renewal ability, which was further confirmed by the creation of germline chimera embryos [85].

### 4.3. Duck

There is still little information on the in vitro culture of duck PGCs, and the truly long-term in vitro culture system has not been established yet, although there have been experiments exploring the subject.

PGCs were successfully isolated from the embryonic gonads of Beijing ducks (*Anas domestica*) at stage HH28, specifically from 7 days of incubation. When co-cultured with mouse embryonic fibroblasts, these duck PGCs could proliferate for more than a month in repeated subcultures and maintain a diploid karyotype. Interestingly, these duck embryonic germ cells (EG) could form embryoid bodies and differentiate into osteoblasts in vitro. The medium they used contained human stem cell factor (SCF), murine leukemia inhibitory factor (LIF), and human basic fibroblast growth factor (bFGF). The cultured PGCs formed colonies over approximately 3 to 7 days. [86].

More recently, another study investigated Muscovy ducks (*C. moschata*), Pekin ducks (*A. platyrhynchos*), and mule ducks, and isolated PGCs from both the circulatory system and the gonads. Duck PGCs could be kept in FAot (FGF2, Activin A, and ovotransferrin) and FAcs (FGF2, Activin A, and chicken serum) media for a limited period. Both circulating and gonadal duck PGCs proliferated better in FAot medium than in FAcs medium and formed fewer aggregates. Muscovy duck circulating PGCs ceased proliferation after approximately 50 days of subculture, while Muscovy duck gonadal PGCs stopped proliferating after just one week in culture. To achieve long-term in vitro culture of duck PGCs, the culture conditions need improvement, potentially by supplementing the medium with additional factors [70].

### 4.4. Zebra Finch

The zebra finch (*Taeniopygia guttata*) is an important model organism in scientific research, particularly in the fields of neuroscience, genetics, behavior, and evolutionary biology [96,97]. The zebra finch can serve as a valuable model for studying wild bird populations. Unlike domesticated species, zebra finches retain natural behaviors such as mate choice, song learning, and territoriality, yet they breed readily in captivity, allowing controlled experimental studies. Research on this species provides important insights into how environmental changes influence avian biology and offers implications for the conservation of other species facing similar ecological challenges. Therefore, to preserve their genetic material, it is essential to establish an in vitro PGC culture for zebra finches [49]. Beyond chickens, zebra finches likely have the most thoroughly characterized PGCs among birds; nevertheless, protocols for long-term in vitro culture similar to those developed for chickens and geese have yet to be established.

We know that the PGC number in blastoderm and gonad is higher in zebra finch than in chicken [87], and gene expression differences were also described, notably finch PGCs show more active Insulin and Nodal signaling pathways while FGF2 is more active in chicken PGCs [98,99]. Another study identified two different PGC populations in HH25 finch gonads, which is surprising since that is not the case in chickens. One population showed strong upregulation of pluripotency genes (NANOG, PRDM14, KIT) while the other population highly expressed genes connected with germ cell fate (FOXL2L) [79].

In an attempt to culture, PGCs from zebra finches were isolated from the embryonic gonads at stage HH28. For each culture, approximately 3–5 pairs of gonads were used. An interesting approach was used when somatic cells from the gonads adhered to the bottom of the plate and acted as a feeder layer while the PGCs remained in suspension. PGCs and feeder cells were subcultured at 4- to 5-day intervals for up to 30 days [87].

Jung et al. employed a similar protocol for the primary culture of zebra finch PGCs, aiming to enhance germline transmission capacity by using a busulfan-mediated system for PGC transplantation into adult testes, with the goal of producing germline chimeras [100]. They have developed primordial germ cells overexpressing microsomal glutathione-S-transferase II (MGSTII), conferring resistance to busulfan, a compound that induces germ cell-specific cytotoxicity. These MGSTII-overexpressing PGCs were transplanted into testes that had been temporarily rendered infertile by an initial dose of busulfan. Following transplantation, recipients were administered a second dose of busulfan to further deplete endogenous germ cells and enhance the integration of transplanted cells, resulting in successful spermatogenesis derived from donor cells. This approach presents a promising method for producing transgenic zebra finches, as it requires fewer recipients due to higher survival rates and eliminates the need to wait for founder maturation, which is necessary when transplanting PGCs into embryos.

### 4.5. Quail

Quails are among the economically valuable farmed animals and have been extensively utilized as avian models in research [101,102].

A recent study showed a cultivation system for pregonadal PGCs isolated from various sources such as embryonic blood, whole embryonic tissues, tail bud tissues, and mixtures of blood and tail bud tissues. The system requires the presence of specific embryonic cells (giant feeder cells) to serve as a feeder layer, which are important for PGC proliferation [71]. Specifically, a complete KAv-1 medium [103] supplemented with 5% KSR was utilized. The medium was replaced with 50% of the total volume regularly every other day, or adjusted based on the color change in the medium. For passaging, gentle mechanical pipetting was employed along with a cGMP enzyme-free cell dissociation reagent to dissociate the cultured cells. Although the culturing was 4–5 months long, it is still a feeder-based technique, and the stable production of these giant cells is questionable [71].

Another study used quail embryonic fibroblasts (QEF) as a feeder and isolated PGCs from gonads and blood. PGCs from the gonad proliferated better than those from the blood. Notable components of the medium were buffalo rat liver (BRL)-conditioned Knockout DMEM, FBS, chicken serum recombinant murine SCF, and recombinant human bFGF. The cells were suspended in 500 μL of PGC culture medium and plated into a well of a 24-well plate to isolate PGCs from somatic cells using the differential adhesion method. After 3–4 h, the floating cells, which predominantly consisted of PGCs, were transferred to a well of a 48-well plate containing a feeder layer of mitomycin-inactivated QEF [88].

### 4.6. Red Junglefowl and Indigenous Pheasants

Red junglefowl and pheasants are recognized as wild avian species that are challenging to manage and therefore need protection to prevent their extinction and maintain avian biodiversity.

In vitro culture of PGCs from these avian species was successful using the KAv-1 medium. Embryos at stage 12 were used for PGC isolation. PGCs were isolated from both blood and embryonic tissues. Circulating PGCs were collected by initially drawing blood from the sinus terminalis and omphalomesenteric artery using a microcapillary until cardiac arrest occurred. Blood was then drawn from the remaining vessels until the embryo was visibly exsanguinated. PGC purification was performed using Nycodenz density gradient centrifugation at 4 °C [32,104]. The purified PGCs were seeded onto inactivated quail feeder cells. Passaging was performed when the PGCs had formed multiple colonies and spread throughout the well, by detaching the PGCs via pipetting and transferring them to a new well containing inactivated quail feeder cells [32].

After blood removal, the remaining embryo was finely disaggregated using a sterile surgical blade, suspended in 1 mL of complete KAv-1 medium, seeded into a 25 cm^2^ flask, and incubated at 38 °C. The flask was tilted during the first week to restrict cell growth to a limited area. Fresh medium (1 mL) was added daily, and subsequently, 50% of the medium was replaced with fresh complete medium. PGCs that proliferated in culture, as indicated by the growth of colonies, were termed “tissue PGCs.” These tissue PGCs originated from the primary culture of the remaining embryonic tissue after most circulating PGCs and blood cells had been removed. The tissue PGC population included residual circulating PGCs, interstitial migrating PGCs, and gonadal PGCs. Cultivating these tissue PGCs maximizes the use of embryonic tissue, which is particularly valuable when working with embryonated eggs from wild species, which are often limited in quantity and variable in quality, especially if the embryos do not reach the gonadal development stage [32]. These embryonic cells and tissue PGCs were collectively passaged and cryopreserved upon reaching confluence, using dissociation by pipetting or a chemical reagent. Both isolated circulating PGCs and tissue PGCs proliferated in the culture media; however, the culture conditions were more conducive to the growth of circulating PGCs from red junglefowl (RJF) compared to those from pheasant species. Further investigations are needed to enhance the proliferation efficiency of both circulating and tissue PGCs from these wild avian species [32].

## 5. Discussion

The results reviewed here underline the importance of primordial germ cells (PGCs) as a versatile tool for avian biotechnology, conservation, and developmental biology. The ability to isolate, propagate, and genetically modify PGCs in vitro has effectively bridged the gap between avian germline biology and practical biotechnological applications. However, despite considerable progress—especially in the chicken model—significant interspecies variability remains a key limitation and a challenge for future research.

The development of long-term, feeder-free culture systems in chickens has provided a robust foundation for understanding the molecular mechanisms of germline self-renewal and pluripotency. Defined media incorporating FGF2, Activin A/BMP, and Insulin signaling [23] have eliminated much of the variability associated with feeder-based and serum-containing systems, and recent advances such as 3D culture environments and serum-free formulations have demonstrated that PGCs can be expanded efficiently without compromising germline competency. Nonetheless, even within chicken breeds, variability in metabolic and nutritional preferences [27] suggests that culture requirements are influenced by subtle genetic and epigenetic differences, challenging the notion of a universal avian PGC medium.

Extending these protocols beyond the chicken has proven difficult. Goose PGCs, for instance, require BMP4 for self-renewal and are inhibited by Activin A [85], indicating a reversed response to TGF-β family signals relative to chicken. Duck and quail PGCs can proliferate for limited periods under chicken-optimized conditions but fail to achieve long-term stability [70,88], highlighting the need for species-specific optimization of signaling components. The divergence of PGC signaling dependencies across taxa likely reflects evolutionary adaptations to embryonic development timing, metabolic context, and environmental pressures.

From an applied perspective, PGC technology has already demonstrated its potential in transgenesis, recombinant protein production, and the preservation of endangered breeds. The establishment of PGC cryobanks circumvents the limitations of avian gamete and embryo cryopreservation, offering a practical route to safeguard both nuclear and mitochondrial genetic information. Furthermore, genome editing in cultured PGCs using CRISPR/Cas9 and related tools has enabled the creation of transgenic lines with disease resistance or altered productivity traits [25,56]. Yet, the transition from laboratory-scale experimentation to scalable, industrial or conservation use will require standardized culture systems, validated cryopreservation protocols, and clear regulatory frameworks governing the use of germline-modified animals.

Future work should focus on integrating molecular, biochemical, and systems-level approaches to further understand and explore the signaling networks that govern PGC fate. High-throughput screening of growth factors and small molecules, combined with transcriptomic profiling, may identify universal core pathways complemented by species-specific modifiers. Furthermore, interdisciplinary collaboration between developmental biologists, geneticists, and conservationists will be essential to translate laboratory findings into sustainable applications for biodiversity preservation and agricultural innovation.

## 6. Conclusions

The in vitro culture of avian primordial germ cells has become a cornerstone of modern avian biotechnology. Progress achieved in chickens has demonstrated that PGCs can be maintained, genetically engineered, and reintroduced to produce germline chimeras, enabling applications in transgenesis, gene conservation, and agricultural improvement. Nevertheless, extending this success to other bird species reveals substantial biological diversity, particularly in growth factor requirements and signaling pathways governing self-renewal. These differences emphasize the need for species-specific optimization and systematic investigation of PGC biology across avian taxa. Future advances will rely on integrating molecular profiling, high-throughput screening, and interdisciplinary collaboration to develop robust, feeder-free, and defined culture systems. Such efforts will not only enhance our understanding of germline biology but also provide sustainable solutions for biodiversity preservation, poultry breeding, and biotechnological innovation.

## Figures and Tables

**Table 1:** Summary of the PGC culture and maintenance results achieved so far in different avian species.

Species	Medium/Base	Key Growth Factors	Key Supplements	Feeder System	Culture Length	Reference(s)
Chicken	KO-DMEM medium conditioned on BRL cells	SCF, FGF2	FCS, chicken serum	STO or BRL cells	~9 months	[82]
	KO-DMEM medium conditioned on BRL cells	SCF, FGF2, LIF	FBS, chicken serum	STO or BRL or CEF cells	~8 months	[83]
	Avian KO-DMEM basal medium (250 mOsm/kg, calcium chloride free)	FGF2, Activin A, Insulin (from B-27 supplement)	B-27 supplement, ovotransferrin, ovalbumin	Feeder-free	~ 8 months (indefinitely)	[23]
	Avian KO-DMEM basal medium (250 mOsm/kg, calcium chloride free)	FGF2, Activin A, Insulin (from B-27 supplement)	B-27 supplement, ovotransferrin, ovalbumin +Knock-Out Serum Replacement (KSR)	Feeder-free	~4 months	[84]
Goose	Avian KO-DMEM basal medium (250 mOsm/kg, calcium chloride free)	FGF2, BMP4, FGF1, IGF1	B-27 supplement, ovotransferrin, ovalbumin +cholesterol, B12	Feeder-free	~4 months (indefinitely)	[85]
Duck	DMEM	SCF, FGF2, LIF	FBS	MEF	~1 month (7 passages)	[86]
	Avian KO-DMEM basal medium (250 mOsm/kg, calcium chloride free)	FGF2, Activin A, Insulin (from B-27 supplement)	B-27 supplement, ovotransferrin, ovalbumin with or without chicken serum	Feeder-free	max. 52 days	[70]
Zebra Finch	KO-DMEM	FGF2	FBS, chicken serum	Gonadal Stromal Cells (GSCs)	~1 month	[87]
	KO-DMEM	BMP4, FGF2, IGF	B-27 supplement, ovalbumin +FBS, choloesterol	Feeder-free	~1 month	[49]
Quail	KO-DMEM medium conditioned on BRL cells	SCF, FGF2, IDE1	Chicken Serum, FBS	Quail Embryonic Fibroblasts (QEF)	~1.5 months	[88]
	KAv-1	Non added	Chicken Serum, FBS +Knock-Out Serum Replacement (KSR)	Autogenic giant multinucleated cells	~5 months	[71]
Pheasants	KAv-1	Non added	FBS	Autogenic embryonic cells	~6 months	[32]

## Data Availability

No new data were created or analyzed in this study. Data sharing is not applicable.

## Abbreviations

The following abbreviations are used in this manuscript:

Activin A	Member of the TGF-β superfamily involved in cell differentiation and proliferation
AlbuMAX	Lipid-rich bovine serum albumin supplement
ANP32	Acidic nuclear phosphoprotein 32 family
bFGF	Basic fibroblast growth factor
BMP4	Bone morphogenetic protein 4
BRL	Buffalo rat liver (cell line used as feeder layer)
cPGC	Circulating primordial germ cell
CRISPR/Cas9	Clustered Regularly Interspaced Short Palindromic Repeats/CRISPR-associated protein 9
cGMP	Cyclic guanosine monophosphate
DMEM	Dulbecco’s Modified Eagle Medium
EG	Embryonic germ (cell)
EG&K	Eyal-Giladi and Kochav stage (chicken blastoderm staging system)
FAot	FGF2, Activin A, and ovotransferrin-based medium
FAcs	FGF2, Activin A, and chicken serum-based medium
FGF1/FGF2	Fibroblast growth factor 1/Fibroblast growth factor 2
FFC	Feather follicle cell
FP003	Functional polymer used in serum-free 3D culture
GRC	Germline-restricted chromosome
gPGC	Gonadal primordial germ cell
GSK-3	Glycogen synthase kinase 3
HH	Hamburger and Hamilton (chicken embryonic development staging system)
hIFNα 2a	Human interferon alpha 2a
HBsAg	Hepatitis B virus surface antigen
H&E	Hematoxylin and eosin (implied histological stain)
IGF-1	Insulin-like growth factor 1
iPSC	Induced pluripotent stem cell
KAv-1	Kuwana Avian medium 1 (PGC culture medium)
KOSR	KnockOut Serum Replacement
LIF	Leukemia inhibitory factor
MGSTII	Microsomal glutathione-S-transferase II
MSC	Mesenchymal stem cell
QEF	Quail embryonic fibroblast
RJF	Red junglefowl
ROS	Reactive oxygen species
SCF	Stem cell factor
SCF2	Membrane-bound form of chicken stem cell factor
SMAD	Intracellular proteins that transduce extracellular TGF-β signals
SDS mating	Sire Dam Surrogate mating
STO	SIM mouse embryo-derived thioguanine-resistant fibroblast cell line
tPGC	Tissue primordial germ cell
TGF-β	Transforming growth factor beta
ZW/ZZ	Avian sex chromosome systems (female ZW, male ZZ)
